# Variation in academic neurosurgery departments’ #neurosurgery social media influence

**DOI:** 10.1016/j.wnsx.2023.100232

**Published:** 2023-06-22

**Authors:** Michael B. Cloney, Benjamin Hopkins, Anastasios Roumeliotis, Najib El Tecle, Nader S. Dahdaleh

**Affiliations:** Feinberg School of Medicine of Northwestern University, Chicago, IL, United States

**Keywords:** Academic neurosurgery, Neurosurgery, Influencer, Social media, Facebook, Instagram, Twitter

## Abstract

**Background:**

Social media use is increasingly common among academic neurosurgery departments, but its relationship with academic metrics remains underexamined.

**Methods:**

We examine the relationship between American academic neurosurgery departments’ number of followers on Twitter, Instagram, and Facebook and the following academic metrics: Doximity Residency rankings, US News & World Report rankings (USNWR) of their affiliated medical schools, and the amount of NIH funding of those schools.

**Results:**

Few departments had disproportionate number of followers. A greater proportion of programs had Twitter accounts (88.9%) than had Instagram (72.2%) or Facebook (51.9%) accounts (p=0.0001). Programs identified as "Influencers" had more departmental NIH funding (p=0.044), more institutional NIH funding (p=0.035), better Doximity residency rankings (p=0.044), and better affiliated medical school rankings (p=0.002). Number of Twitter followers had the strongest correlation with academic metrics, yet only modest correlations were identified to departmental NIH funding (R=0.496, p=0.0001), institutional NIH funding (R=0.387, p=0.0072), Doximity residency rank (R=0.411, p=0.0020), and affiliated medical school ranking (R=0.545,p<0.0001). On multivariable regression, only being affiliated with a medical school in the top quartile on the USNWR rankings, rather than neurosurgery departmental metrics, predicted having more Twitter (OR=5.666, p=0.012) and Instagram (OR=8.33, p=0.009) followers.

**Conclusion:**

American academic neurosurgery departments preferentially use Twitter over Instagram or Facebook. Their Twitter or Instagram presences are associated with better performance on traditional academic metrics. However, these associations are modest, suggesting that other factors contribute to a department’s social media influence. A department’s affiliated medical school may contribute to the department’s social media brand.

## 1 Background

Social media use in neurosurgery is expanding rapidly, with an exponential increase in the number of neurosurgery departmental social media accounts between 2019 and 2021.^1^ Nearly all neurosurgery trainees use social media, and some experienced users have published guidelines for neurosurgeons to build social media presences.^2^^,^^3^ Despite a rapid increase in social media use among academic neurosurgery departments, neurosurgery departments' social media metrics, and their relationship with traditional academic metrics, remain underexamined. Early studies demonstrated modest correlations between academic productivity and social media usage.^4^ In the intervening years, the use of social media in academic neurosurgery has expanded rapidly, with the number of academic neurosurgery department social media accounts increasing severalfold.^1^^,^^4^ Therefore, a reassessment of social media's relationship to traditional metrics of success in academic neurosurgery are warranted.^5^

## 2 Methods

We identified the American academic neurosurgery departments with the most NIH funding, as determined by the Blue Ridge Institute for Medical Research 2021 edition (BRIMR), obtained in May of 2022. BRIMR is an independent, non-profit, scientific research institute that has monitored NIH funding to health science schools and organizations since 2006.^6^, ^7^, ^8^ Further data was then collected on the 54 departments identified.

Residency rankings were taken from Doximity.com's 2022 Residency Navigator reputation ranking.^9^^,^^10^ Affiliated medical school rankings and institutional NIH funding amounts were determined from the US News & World Report 2023 Best Medical Schools: Research rankings (USNWR rank).^11^ For the purposes of geographic comparison, each program's geographic location was used to classify it into regions, and region divisions, as defined by the US Census Bureau (Table 1).^12^

**Table 1:** Program and academic data among the 54 BRIMC ranked neurosurgery programs.

	n	%
**Programs with Twitter**	48	88.9%
Influencer	27	50.0%
**Programs with Instagram**	39	72.2%
**Programs with Facebook**	28	51.9%
**Programs USNWR Rankings**	49	90.7%
**Programs by Region**		
Northeast	12	22.2%
South	12	22.2%
Midwest	19	35.2%
West	10	18.5%
	**Median**	**IQR**
**Departmental NIH Funding**	$ 1,865,744	[670519, 4505808]
**Institutional NIH Funding**	$ 287,473,010	[146014865, 525076407]
**Medical School Rank**	33.5	56,14
**Doximity Rank**	36	64,16

For each neurosurgery department ranked in the BRIMR ranking, we collected data on whether the department had a social media account, and their number of followers, for the following three social media platforms: Twitter, Instagram, and Facebook. Among departments that had Twitter accounts, their number of followers was retrieved from the account's Twitter page on May 24, 2022. Among departments that had Instagram accounts, their number of followers was retrieved on April 24, 2023. Among departments that had Facebook pages, their number of followers was retrieved on April 25, 2023.

Influencer status was determined by RightRelevance.com, as has been described in past literature.^13^, ^14^, ^15^, ^16^ RightRelevance.com uses a pagerank-like algorithm to find the relative rank of Influencers within a given topic, and calculates a normalized score representing the impact that an influencer has on the community surrounding a specific topic on Twitter. The influencer score, for each department for the topic “Neurosurgery” was recorded. Any department that qualified to have an influencer score was considered an Influencer. Departments that did not have Twitter accounts, and departments with Twitter accounts that were unranked with respect to influencer score, were not considered Influencers. Influencer scores were obtained on the 24^th^ of May 2022. Influencer status and number of social media followers will together be referred to as social media presence hereafter.

For analysis, the programs identified were categorized into quartiles with respect to their number of social media followers. These quartiles were then compared with respect to their academic metrics (amount of departmental NIH funding, amount of institutional NIH funding, Doximity Residency rank, and USNWR rank). Data was stored and managed with Microsoft Excel version 16.4 (Microsoft, Redmond, WA, USA). Statistical analysis was performed using Prism 9.0 (GraphPad Software, Inc., La Jolla, CA, USA) and Stata 12.0, and data visualization was performed using Prism 9.0.

The decision to use parametric or non-parametric assumptions was based on assessing the kurtosis and skewness of the data. Normally distributed data was reported as mean ​± ​standard deviation and compared using ANOVA or *t-*tests. Non-normal data was reported as median [25^th^ percentile, 75^th^ percentile] for nonbinary variables, and compared using Mann–Whitney *U* for ordinal or continuous variables, or Fisher's Exact tests for binary variables. Odds ratios or Hodges-Lehmann differences between medians, and their 95% confidence intervals, were reported as appropriate.

Simple linear regression analysis was performed to identify correlations between each departments number of social media followers and traditional academic metrics. Slope, correlation coefficient R, and *p*-values were reported. The 95% confidence intervals for the best-fit line were depicted on the associated figures.

Stepwise, backward multivariable logistic regression was performed with candidate variables to identify predictors of being in the top quartile of programs with respect to number of social media followers, and of having Influencer status. Candidate variables with *p* ​< ​0.20 on univariable analysis were retained in the multivariable model, and a value of *p* ​< ​0.05 was considered statistically significant on multivariable analyses. For multivariable regression analysis, the number of followers was scaled by a factor of 0.01, so that the odds ratios calculated by the regression models reflects the odds of the outcome of interested associated with an increase of 100 followers.

## 3 Results

**Fig. 1:**
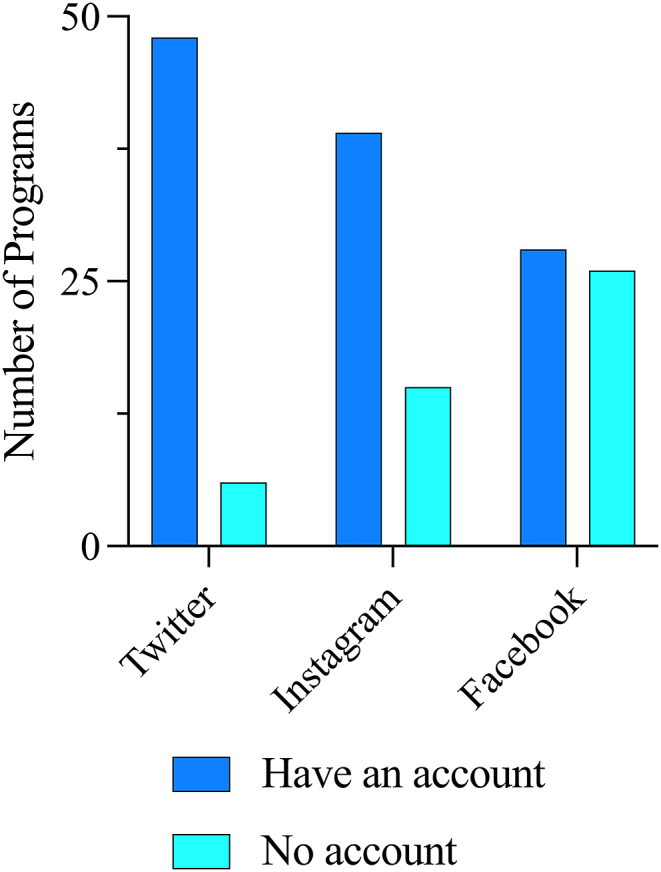
There is a statistically significant difference in the proportion of programs using each social media platform (*p* ​= ​0.0001). A significantly greater number of programs had Twitter accounts (*n* ​= ​48) than Instagram accounts (*n* ​= ​39) (88.9% vs. 72.2%, OR ​= ​3.08 [1.13, 8.68], *p* ​= ​0.050), and a greater number had Instagram accounts than Facebook accounts (*n* ​= ​28) (72.2% vs. 51.9%, OR-2.41 [1.08, 5.32], *p* ​= ​0.0468).

**Fig. 2:**
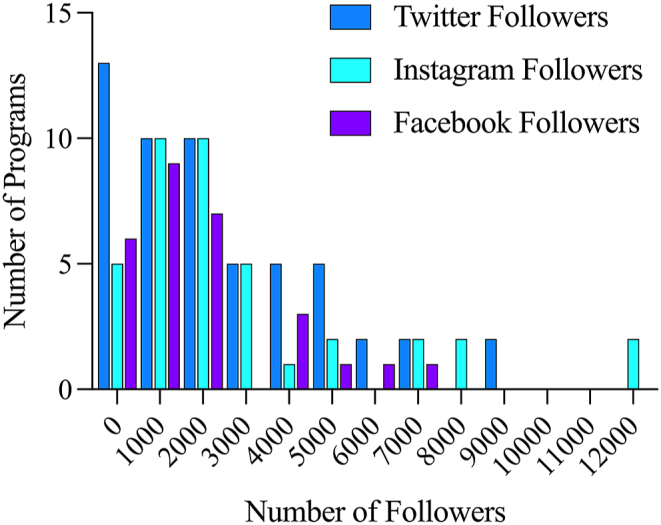
The distribution of the number of social media followers among American academic neurosurgery programs is highly skewed (skewness ​= ​1.059 for Twitter, 1.880 for Instagram, and 1.439 for Facebook), with a few departments having a disproportionate number of followers.

*Descriptive Statistics*. Fifty-four programs were included in the latest BRIMR NIH funding rankings for neurosurgery. The median amount of departmental NIH funding per department was $1,865,744, and the median amount of institutional NIH funding per department was $287,473,010 (Table 1). The median USNWR medical school rank was 33.5, and the median Doximity rank was 36. There is a statistically significant difference in the proportion of programs using each platform (*p* ​= ​0.0001, Fig. 1). A significantly greater number of programs had Twitter accounts (*n* ​= ​48) than Instagram accounts (*n* ​= ​39) (88.9% vs. 72.2%, OR ​= ​3.08 [1.13, 8.68], *p* ​= ​0.050), and a greater number had Instagram accounts than Facebook accounts (*n* ​= ​28) (72.2% vs. 51.9%, OR-2.41 [1.08, 5.32], *p* ​= ​0.0468). The median number of followers for Twitter, Instagram, and Facebook were 1845, 1753, and 1214, respectively (Table 1). The number of followers per department was highly skewed, with a few departments having a disproportionate number of followers (skewness ​= ​1.059 for Twitter, 1.880 for Instagram, 1.439 for Facebook, Fig. 2).

## 4 Followers

**Fig. 3:**
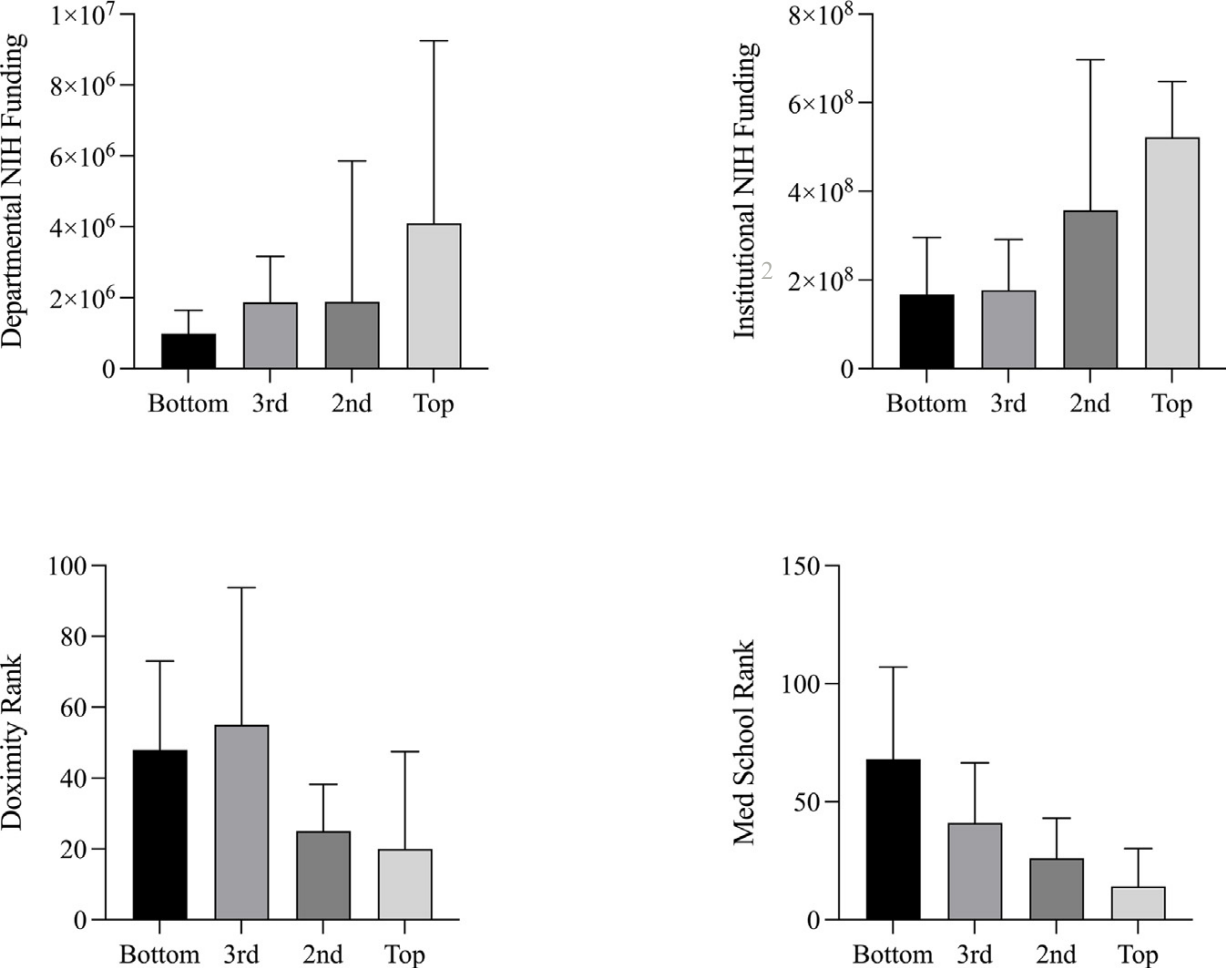
Comparing the academic metrics for American academic neurosurgery departments when departments are stratified into quartiles with respect to their number of Twitter followers. Differences are statistically significant in all cases (*p* ​< ​0.05).

Programs in the top quartile with respect to their number of Twitter and Instagram followers had more departmental NIH funding (*p* ​= ​0.0392 and *p* ​= ​0.011, respectively), more institutional NIH funding (*p* ​= ​0.0082 and *p* ​= ​0.020, respectively), better Doximity residency rankings (*p* ​= ​0.0064 and *p* ​= ​0.011, respectively), and better affiliated medical school rankings (*p* ​= ​0.0004 and *p* ​< ​0.001, respectively) (Fig. 3).

Programs in the top quartile with respect to their number of Facebook were no different from programs that were not with respect to their amount of departmental NIH funding (*p* ​= ​0.640), institutional NIH funding (*p* ​= ​0.452), Doximity residency rankings (*p* ​= ​0.381), affiliated medical school rankings (*p* ​= ​0.097).

## 5 Twitter influencers status

**Fig. 4:**
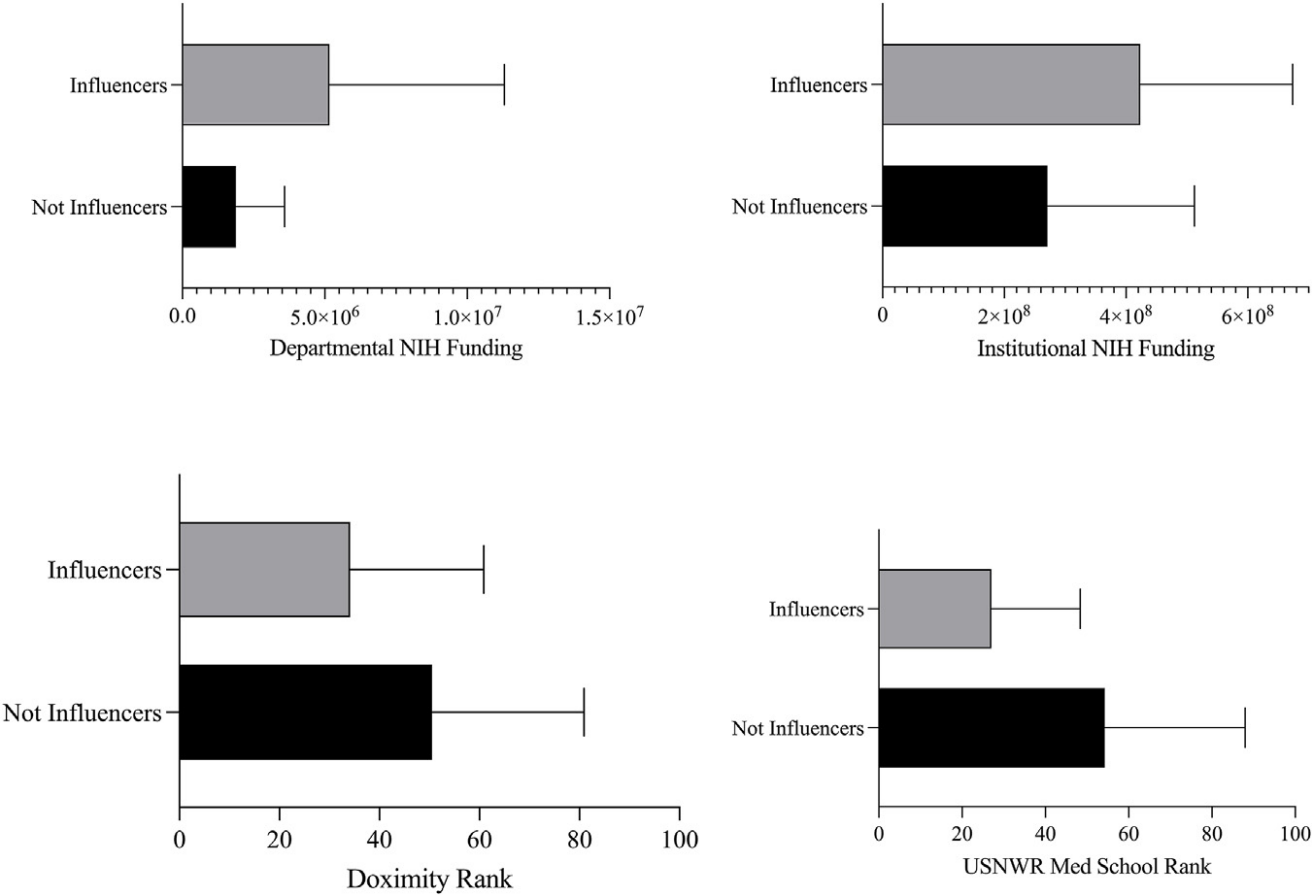
Comparing academic metrics between American academic neurosurgery departments after stratification by their Twitter influencer status. Differences are statistically significant in all cases (*p* ​< ​0.05).

Among the 54 programs examined, 27 (50%) were identified as “Influencers” by the presence of a Twitter profile and a RightRelevance.com neurosurgery category rank score (Table 2). Programs identified as Influencers had more departmental NIH funding (*p* ​= ​0.044), more institutional NIH funding (*p* ​= ​0.035), better Doximity residency rankings (*p* ​= ​0.044), and better affiliated medical school rankings (*p* ​= ​0.002) (Table 3, Fig. 4). Program region was not associated with number of followers (*p* ​= ​0.2039) or influencer status (*p* ​= ​0.3387).

**Table 2:** Top NIH funded programs with influencer status.

Program (alphabetic order)	Number of Twitter Followers
BAYLOR COLLEGE OF MEDICINE	2218
DUKE UNIVERSITY	7211
EMORY UNIVERSITY	2925
ICAHN SCHOOL OF MEDICINE MOUNT SINAI	8844
JOHNS HOPKINS UNIVERSITY	4168
NEW YORK UNIVERSITY SCHOOL OF MEDICINE	780
NORTHWESTERN UNIVERSITY CHICAGO	4021
OREGON HEALTH & SCIENCE UNIVERSITY	6249
STANFORD UNIVERSITY	6944
STATE UNIVERSITY OF NEW YORK BUFFALO	2347
UNIVERSITY OF CALIFORNIA LOS ANGELES	2288
UNIVERSITY OF CALIFORNIA SAN FRANCISCO	8634
UNIVERSITY OF CINCINNATI	1937
UNIVERSITY OF FLORIDA	1672
UNIVERSITY OF KENTUCKY	1627
UNIVERSITY OF MARYLAND BALTIMORE	1752
UNIVERSITY OF MIAMI SCHOOL OF MEDICINE	3678
UNIVERSITY OF MICHIGAN	4679
UNIVERSITY OF MINNESOTA	3901
UNIVERSITY OF OKLAHOMA HLTH SCIENCES CTR	3617
UNIVERSITY OF PENNSYLVANIA	2679
UNIVERSITY OF PITTSBURGH	4846
UNIVERSITY OF SOUTH FLORIDA	1673
UNIVERSITY OF TEXAS HLTH SCI CTR SAN ANTONIO	4869
UNIVERSITY OF VIRGINIA	2454
WEILL MEDICAL COLLEGE OF CORNELL UNIVERSITY	2165
YALE UNIVERSITY	4563

**Table 3:** Comparison between influencers and non-influencers with respect to academic metrics.

	**Influencers**	**Not Influencers**	**Δ**	**95% CI**	***p***
**Departmental NIH Funding**	$2,620,553	$1,250,206	$1,422,854	[34308, 3581901]	0.044
**Institutional NIH Funding**	$465,747,457	$210,868,572	$186,976,327	[11585257, 333635653]	0.035
**Median USNWR Medical School Ranking**	22	41	24	[9, 39]	0.002
**Median Doximity Residency Ranking**	21	48	17	[1, 33]	0.044

Programs that were influencers had significantly more Instagram followers than those that were not (3617 vs. 562, *p* ​< ​0.001), but were no different with respect to their median number of Facebook followers (*p* ​= ​0.212). There is a weak correlation between a program's number of Twitter followers and its number of Instagram followers (R ​= ​0.4859, *p* ​= ​0.0027) and Facebook followers (R ​= ​0.5427, *p* ​= ​0.0051), and no correlation between its number of Instagram followers and number of Facebook followers (R ​= ​0.1128).

**Fig. 5:**
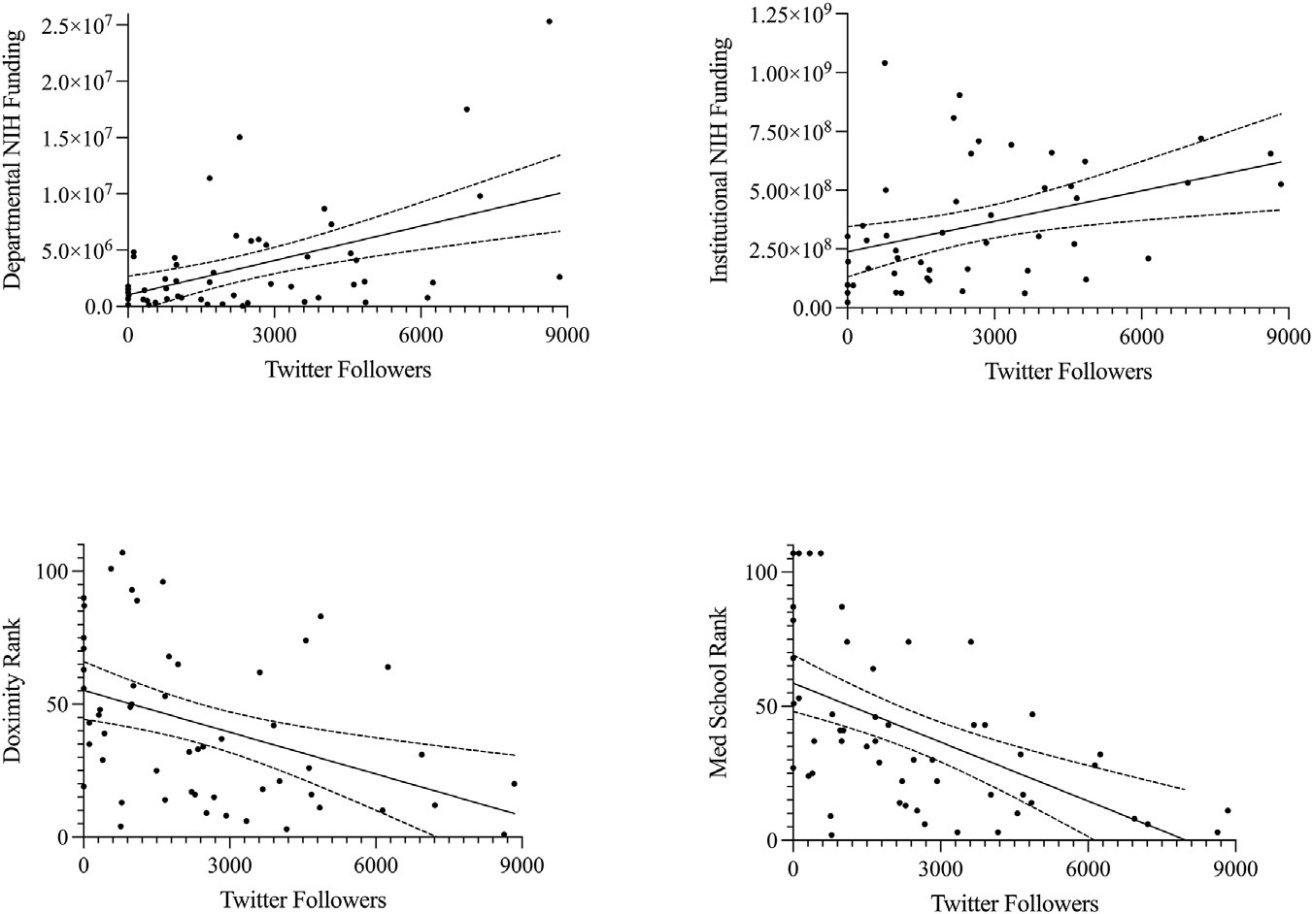
Linear regression directly comparing the number of Twitter followers for American academic neurosurgery departments when departments. Regressions are statistically significant in all cases (*p* ​< ​0.05), with modest correlations.

*Correlations between academic metrics and social media presence*. Modest correlations were detected between a department's number of Twitter followers and its departmental NIH funding (R ​= ​0.496, *p* ​= ​0.0001), institutional NIH funding (R ​= ​0.387, *p* ​= ​0.0072), Doximity residency rank (R ​= ​0.411, *p* ​= ​0.0020), and affiliated medical school ranking (R ​= ​0.545, *p* ​< ​0.0001) (Table 4, Fig. 5).

**Table 4:** Correlations between number of followers and academic metrics.

	Slope	R	*p*	Correlation
Twitter				
**Departmental NIH Funding**	2.41E-04	0.496	0.0001	Weak
**Institutional NIH Funding**	3.47E-06	0.387	0.0072	Weak
**USNWR Medical School Ranking**	−40.5	0.545	<0.0001	Moderate
**Doximity Residency Ranking**	−32.2	0.411	0.0020	Weak
**Instagram**				
**Departmental NIH Funding**	2.71E-04	0.500	0.0012	Moderate
**Institutional NIH Funding**	3.48E-06	0.298	0.0865	None
**USNWR Medical School Ranking**	−44.9	0.481	0.0019	Weak
**Doximity Residency Ranking**	−26.9	0.273	0.0918	None
**Facebook**				
**Departmental NIH Funding**	4.19E-05	0.141	0.4743	None
**Institutional NIH Funding**	1.57E-06	0.204	0.3492	None
**USNWR Medical School Ranking**	−20.2	0.380	0.0457	Weak
**Doximity Residency Ranking**	−18.4	0.313	0.1041	None

Modest correlations were detected between a department's number of Instagram followers and its departmental NIH funding (R ​= ​0.500, *p* ​= ​0.0012) and affiliated medical school ranking (R ​= ​0.4820, *p* ​= ​0.0019), but not its institutional NIH funding (R ​= ​0.2984, *p* ​= ​0.0865), or Doximity residency rank (R ​= ​0.2737, *p* ​= ​0.0918) (Table 4).

There was a weak correlation between a department's number of Facebook followers and its affiliated medical school ranking (R ​= ​0.3807, *p* ​= ​0.0457), but not its departmental NIH funding (R ​= ​0.1410), its institutional NIH funding (R ​= ​0.2045), or its Doximity residency ranking (R ​= ​0.3137, *p* ​= ​0.1041) (Table 4).

**Fig. 6:**
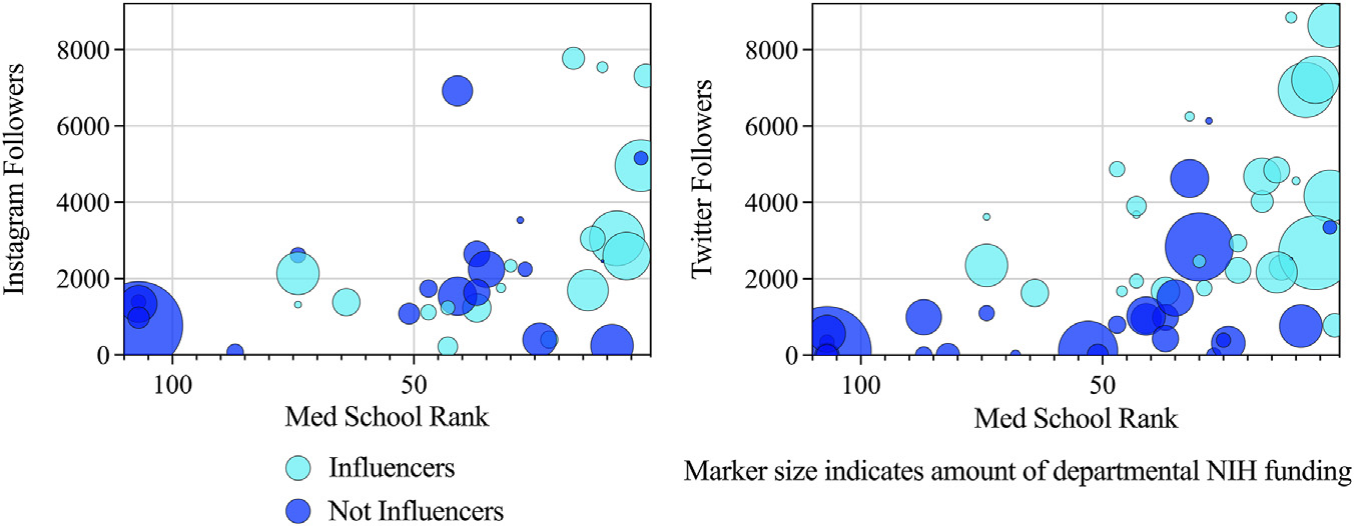
Bubble plots depicting the relationship between medical school rank, number of Twitter or Instagram followers, and amount of NIH funding among American academic neurosurgery departments. No programs fell within the upper left quadrant of the graph, indicating that no programs with low medical school ranks had a high number of Twitter or Instagram followers.

*Independent Associations with Number of followers and influencer status*. On multivariable regression, only being affiliated with a medical school in the top quartile on the USNWR rankings, rather than neurosurgery departmental metrics, predicted being among the top quartile of programs with respect to Twitter followers (OR ​= ​5.666 [1.453777, 22.08806], *p* ​= ​0.012) and Instagram followers (OR ​= ​8.333 [1.697, 40.911], *p* ​= ​0.009). None of the metrics assessed were independently associated with being in the top quartile with respect to Facebook followers. No programs with a low medical school rank had a high number of Twitter or Instagram followers (Fig. 6).

On multivariable regression, number of Twitter followers (OR ​= ​1.083 [1.031, 1.138], *p* ​= ​0.001), rather than academic metrics or Facebook and/or Instagram followers, was the only factor independently associated with influencer status.

## 6 Discussion

Social media use among academic neurosurgery departments is expanding rapidly, but its relationship to academic metrics is unclear.^1^^,^^17^ Here, we find that departments with more Twitter and Instagram followers have more NIH funding, have more highly ranked residencies, and are affiliated with more highly ranked medical schools. However, this relationship was not observed for Facebook followers. Moreover, academic metrics were more often, and more strongly, correlated with Twitter and Instagram metrics than with Facebook metrics. However, on multivariable analysis, a high number of Twitter or Instagram followers was associated with the ranking of a department's affiliated medical school, rather than metrics specific to the neurosurgery department itself. Moreover, the direct correlation between individual departments' academic metrics and their social media presences was modest, suggesting that differences between neurosurgery departments' social medica presence are only partially due to academic impact or reputation.

Our finding that a department's social media presence was modestly associated with academic metrics is consistent with the growing use of social media by academic neurosurgery departments. In their 2016 article examining social media use among academic neurosurgery departments, Alotaibi et al identified social media accounts representing only 22 of the 119 (18.4%) North American academic neurosurgery departments.^4^ Here, we found that Twitter was the most used social media platform of the three analyzed, and identified Twitter accounts for 48 out of 54 (88.9%) of the top 54 NIH-funded American neurosurgery departments, suggesting a significant increase in Twitter use in recent years,. Indeed, Daggubati et al noted an exponential increase in neurosurgery departmental Twitter accounts between 2019 and 2021. Lamano et al demonstrated that Twitter is second only to YouTube with respect to usage by academic neurosurgery departments, and is the social media platform most used by individual neurosurgeons.^1^^,^^5^

We found that neurosurgery departments with more NIH funding or higher academic rankings had more followers on social media, which is consistent with prior studies that have noted an association between social media presence and academic metrics. Daggubati et al noted that departments with higher Doximity rankings were more likely to have Twitter accounts.^13^, ^14^, ^15^, ^16^ Alotaibi et al found that departments with social media accounts had more publications and citations than departments without them.^4^ Moreover, the relationship between bibliometric success and social media presence extends to neurosurgical journals as well.^18^ Of note, such associations are not explanatory, and further research is needed to better characterize the relationship academic metrics and social media influence.

Our finding that only USNWR rank is independently associated with having more social media followers suggests that institutional academic metrics, rather than departmental ones, may contribute more significantly to a neurosurgery department's social media presence. However, as mentioned above, such associations must be interpreted cautiously. Given the widespread dissemination of USNWR medical school rankings, they may influence perceptions among non-neurosurgeons. For example, USNWR medical school rank, rather than Doximity residency rank, has been shown to be associated with patients' perceptions of their neurosurgeon.^19^ Indeed, Lamano et al concluded that among neurosurgery departments with Twitter accounts, a department's number of Twitter followers, rather than other academically based rankings, was independently associated with composite patient satisfaction scores on physician rating websites.^5^ While such correlations are multifactorial, and conclusions cannot confidently be drawn, these findings are at least consistent with the idea that branding contributes to public perception. Indeed, neurosurgical patients are known to discuss symptoms and treatment options online, and Twitter has proven to be an effective means of disseminating neurosurgical information.^20^^,^^21^^,^^22^

Our finding of a modest correlation between academic strength metrics and a department's number of social media followers, is consistent with existing literature. Riccio et al compared the top 100 neurosurgery influencers on Twitter, and similarly found a modest correlation of R ​= ​0.35 between influencer rank and h-index.^13^, ^14^, ^15^, ^16^ Wang et al have suggested that neurosurgical journals with a greater social media presence have greater outreach and engagement.^18^ These metrics – influencer ranking and h-index – are distinct from the metrics studied here, but nevertheless support the interpretation that while academic output is correlated with social media influence, it is only one of many factors. Indeed, none of the academic metrics assessed were independently associated with Influencer status on multivariable regression.

Daggubati et al, in their analysis of 4962 tweets from academic neurosurgery departments, noted that the topic of nearly half of all tweets (49.9%) was faculty and resident promotion, with disease awareness, advocacy/fundraising, and departmental promotion each ranging from 13.1% to 15.7% of all tweets.^1^ This suggests a social media ecosystem on Twitter in which faculty and trainees follow and support their departments’ accounts, which in turn promote their faculty and trainees. Similarly, Pando et al have recently demonstrated that neurosurgical influencers on Instagram devote 67.4% of their posts to clinical and professional content.^23^ By contrast, Waqas et al found that Facebook was the most commonly used social media platform by neurosurgical trainees (87.1%), but also that most used it for primarily personal rather than academic or professional purposes.^2^

Indeed, in our anlaysis, Facebook was the social media platform with the fewest associations and weakest correlations with academic success. Despite the finding of Waqas et al that Facebook was the most commonly used social media platform by neurosurgical trainees (87.1%),^2^ Lamano et al found academic neurosurgery departments more frequently used Twitter,^5^ which is consistent with our finding that a greater proportion of programs had a Twitter account. Moreover, Wang et al compared social media mentions to Altmetric scores – a measure of the amount of attention a research article has received – and found that Tweets had a greater influence on Altmetric scores than other kinds of social media. Thus, our finding that Twitter and Instagram metrics are better correlated with academic metrics than Facebook is consistent with existing literature on social media use in academic neurosurgery.

Our study has several limitations. While neurosurgical NIH funding is well correlated with publication bibliometrics, it does not capture the multifaceted nature of an academic medical career and academic contribution to a field.^24^ While both are widely known and used, USNWR rankings and Doximity rankings are controversial.^10^^,^^25^ RightRelevance.com has been similarly used elsewhere in published surgical literature, but its method of calculating influencer scores is proprietary and cannot be scrutinized by investigators.^13^, ^14^, ^15^ Moreover, social media by its nature is constantly evolving, and the number of followers and the influencer status of the social media accounts assessed have almost assuredly already changed since our data was collected. While we have successfully identified several associations, our findings are not explanatory, and are limited to being hypothesis generating. Finally, while there is a growing body of literature on the use of social media in neurosurgery, additional studies are required to characterize its current impact, and to determine how optimal use can be best obtained.

## 7 Conclusion

Neurosurgery departments with more departmental NIH funding, institutional NIH funding, or that are ranked more highly tend to have more Twitter and Instagram followers. However, these associations are modest, suggesting that a multitude of factors contribute to a neurosurgery department's social media strategy, and subsequently play a significant role in determining its social media influence. Associations between academic metrics and Facebook metrics are relatively weaker than the associations with Twitter and Instagram metrics. A neurosurgery department's affiliated medical school may contribute to the department's brand on social media.

## CRediT authorship contribution statement

**Michael B. Cloney:** Conceptualization, Data curation, Formal analysis, Methodology, Project administration, Software, Visualization, Writing – original draft, Writing – review & editing. **Benjamin Hopkins:** Data curation, Supervision, Validation, Visualization, Writing – review & editing. **Anastasios Roumeliotis:** Data curation, Project administration, Resources, Validation, Writing – review & editing. **Najib El Tecle:** Conceptualization, Supervision, Validation, Writing – review & editing. **Nader S. Dahdaleh:** Conceptualization, Resources, Software, Supervision, Validation, Writing – review & editing.

## Declaration of competing interest

Dr. Nader S. Dahdaleh is a paid consultant for DePuy Synthes, but has no financial or institutional interest in any of the materials or devices described in this articleThe remainder of the authors have no financial disclosures, and have no personal, financial or institutional interest in any of the materials or devices described in this article.
